# *Bridelia ferruginea* Produces Antineuroinflammatory Activity through Inhibition of Nuclear Factor-kappa B and p38 MAPK Signalling

**DOI:** 10.1155/2012/546873

**Published:** 2012-12-18

**Authors:** Olumayokun A. Olajide, Mutalib A. Aderogba, Uchechukwu P. Okorji, Bernd L. Fiebich

**Affiliations:** ^1^Division of Pharmacy and Pharmaceutical Science, Department of Chemical and Biological Sciences, University of Huddersfield, Queensgate, Huddersfield HD1 3DH, UK; ^2^Neurochemistry Research Laboratory, Department of Psychiatry and Psychotherapy, University of Freiburg Medical School, Hauptstraße 5, 79104 Freiburg, Germany; ^3^Department of Chemistry, Faculty of Science, Obafemi Awolowo University, Ile-Ife, Nigeria; ^4^VivaCell Biotechnology GmbH, Ferdinand-Porsche-Straße 5, 79211 Denzlingen, Germany

## Abstract

*Bridelia ferruginea* is commonly used in traditional African medicine (TAM) for treating various inflammatory conditions. Extracts from the plant have been shown to exhibit anti-inflammatory property in a number of *in vivo* models. In this study the influence of *B. ferruginea* (BFE) on the production of PGE_2_, nitrite, and proinflammatory cytokines from LPS-stimulated BV-2 microglia was investigated. The effects of BFE on cyclooxygenase-2 (COX-2) and inducible nitric oxide synthase (iNOS) protein expressions were evaluated in LPS-activated rat primary microglia. The roles of NF-**κ**B and MAPK signalling in the actions of BFE were also investigated. BFE (25–200 **μ**g) inhibited the production of PGE_2_, nitrite, tumour necrosis factor-**α** (TNF**α**), and interleukin-6 (IL-6) as well as COX-2 and iNOS protein expressions in LPS-activated microglial cells. Further studies to elucidate the mechanism of anti-inflammatory action of BFE revealed interference with nuclear translocation of NF-**κ**Bp65 through mechanisms involving inhibition of I**κ**B degradation. BFE prevented phosphorylation of p38, but not p42/44 or JNK MAPK. It is suggested that *Bridelia ferruginea* produces anti-inflammatory action through mechanisms involving p38 MAPK and NF-**κ**B signalling.

## 1. Introduction

Traditional African medicine (TAM) is a shorthand reference to indigenous forms of healing that are practiced all over Africa [1]. *Bridelia ferruginea* Benth. (Euphorbiaceae) is a shrub which is employed in TAM for treating arthritis and as an embrocation for the treatment of bruises, boils, dislocation, and burns [2, 3]. Tea made from the pulped bark is used for fevers, headaches, stiffness, and rheumatic pains and as a local application for treating oedemas [4]. Earlier, we observed that the aqueous extract of *B. ferruginea* stem bark exhibited inhibition of both the carrageenan-induced rat paw oedema and cotton pellet granuloma formation in rats [5]. Topical anti-inflammatory, antiarthritic, antipyretic, and analgesic properties of the plant were also reported by us [6]. We also showed that a stem bark extract of *B. ferruginea* protected mice from lipopolysaccharide- (LPS-) induced septic shock and inhibited LPS-induced vascular permeability [7]. Recently, Akuodor et al. [8] reported that a stem bark extract of *B. ferruginea* exhibited potential analgesic and antipyretic properties in mice and rats. Extracts from the plant have been reported to demonstrate *in vitro* antioxidant activity in the DPPH free radical scavenging assay [9]. In spite of the varied ethnopharmacological applications of *B. ferruginea* in inflammatory conditions and its demonstrated actions *in vivo*, to our knowledge there is no documented evidence of the molecular target(s) of its anti-inflammatory actions.

Cyclooxygenase (COX) is an enzyme that is required for the synthesis of prostaglandins. There are two subtypes of this enzyme: cyclooxygenase-1 (COX-1) and cyclooxygenase-2 (COX-2). COX-1 is expressed constitutively in most cell types, whereas expression of COX-2 is induced by various factors including inflammatory cytokines and is responsible for the maintenance of the inflammatory process.

Nuclear factor-kappa B (NF-*κ*B) is a transcription factor which has been considered to be the critical central regulator of the inflammatory process. NF-*κ*B controls the expression of genes encoding the proinflammatory cytokines (IL-6, TNF*α*, IL-1, IL-2, etc.), chemokines (IL-8, mcp-1, RANTES, etc.), adhesion molecules (ICAM, VCAM, E-selectin), inducible enzymes (COX-2 and iNOS), growth factors, some acute phase proteins, and immune receptors, all of which play critical roles in controlling major inflammatory processes [10]. NF-*κ*B has therefore become a critical molecular target in drug discovery, and several natural and synthetic compounds have been investigated for their potential to inhibit NF-*κ*B [11].

The mitogen-activated protein (MAP) kinases are intracellular enzymes which allow cells to respond to stimuli such as inflammatory cytokines, from their extracellular environment [12]. MAPKs include extracellular signal-regulated kinases (ERK 1/2), c-Jun N-terminal kinases (JNKs) and p38 isoforms. p38 MAPK pathway has been shown to play a central role in the expression and activity of proinflammatory cytokines such as TNF*α*, IL-6, IL-7, and IL-8 in many cell types [13]. Evidences supporting the importance of the p38 MAPK in inflammatory diseases, such as asthma, rheumatoid arthritis, systemic inflammation, inflammatory bowel disease, and brain inflammation, have been reviewed by Yong et al. [13].

In this study we have used cellular models of CNS inflammation to show that a stem bark extract of *B. ferruginea* (BFE) produces antineuroinflammatory action through inhibition of steps in the signalling of NF-*κ*B and p38 MAPK.

## 2. Materials and Methods

### 2.1. Plant Material

*Bridelia ferruginea* stem bark was collected from a tree growing on the University of Ibadan Campus, Nigeria. The plant samples were identified in the Herbarium, Botany Department, University of Ibadan as well as the Herbarium, Forestry Research Institute of Nigeria, Ibadan, Nigeria. These samples were authenticated using voucher specimens deposited at various periods. A voucher specimen of the collected plant samples was also deposited in the FRIN herbarium, and given the specimen number F.H.I. 106501. The stem bark was air-dried at room temperature, powdered, and extracted in a mixture of methanol and water (1 : 1) for 24 h. The resulting extract was further extracted for additional 24 h. The extract was filtered and concentrated *in vacuo* at 24°C.

### 2.2. Cell Culture

Primary mixed glial cell cultures were established from cerebral cortices of one-day neonatal Sprague-Dawley rats as described earlier [14, 15]. Forebrains were minced and gently dissociated by repeated pipetting in PBS and filtered through a 70 *μ*M cell strainer (Falcon). Cells were collected by centrifugation (1000 g, 10 min), resuspended in Dulbecco's modified Eagle's medium (DMEM) containing 10% fetal calf serum (Biochrom AG, Berlin, Germany) and antibiotics (40 U/mL penicillin and 40 lg/mL streptomycin PAA Laboratories, Coelbe, Germany), and cultured on 10 cm cell culture dishes (5 × 10^5^ cells/plate) in 5% CO_2_ at 37°C. Floating microglia were harvested every week (between 2 and 7 weeks) and reseeded into 75 cm^2^ culture flask to give pure microglial cultures. The following day, cultures were washed to remove nonadherent cells, and fresh medium was added. The purity of the microglial culture was >98% as previously determined by immunofluorescence and cytochemical analysis [14].

BV-2 mouse microglia cell line ICLC ATL03001 (Interlab Cell Line Collection, Banca Biologica e Cell Factory, Italy) was cultured in RPMI 1640 (Gibco) supplemented with 10% FBS (Sigma), 2 mM glutamine (Sigma). Cells were split 1 : 5 when they reached confluence using trypsin/EDTA solution in PBS.

The human neuroblastoma (SK-N-SH) cells were obtained from the HPA Culture Collection (Salisbury, UK) and were grown in MEM-Eagle's medium (Life Technologies, UK), which does not contain any anti-inflammatory substance. Medium was supplemented with 5% foetal bovine serum (Sigma, UK), 2 mM L-glutamine, 1 mM sodium pyruvate, and 40 units/mL penicillin/streptomycin (Sigma, UK). Confluent monolayers were passaged routinely by trypsinisation. Cultures were grown at 37°C in 5% CO_2_ until 80% confluence, and the medium was changed the day before treatment.

### 2.3. PGE_**2**_ Enzyme Immunoassay (EIA)

Quantification of PGE_2_ accumulation was carried out in BV-2 cells by seeding in 96-well plates (2 × 10^5^/200 *μ*L/well), cultured for 2 days, and incubated with or without LPS (100 ng/mL) in the absence or presence of BFE (25–200 *μ*g/mL) for 24 h. PGE_2_ concentration was assessed in cell supernatants with a commercially available kit (Arbor Assays, Ann Arbor, MI, USA), followed by measurement at 450 nm with a microplate reader. Experiments were performed at least three times and in triplicate.

### 2.4. Nitrite Assay

Quantification of nitrite accumulation in BV-2 cells was carried out as described earlier [16]. Cells were seeded in 96-well plates (2 × 10^5^/200 *μ*L/well), cultured for 2 days, and then incubated with or without LPS (100 ng/mL) in the absence or presence of BFE (25–200 *μ*g/mL) for 24 h. As a parameter of NO production, nitrite concentration was assessed in cell supernatants using the Griess assay with a commercially available kit (Promega, Southampton UK), followed by measurement at 540 nm with a Tecan F50 microplate reader. Nitrite concentrations in the supernatants were determined by comparison with a sodium nitrite standard curve. Experiments were performed at least three times and in triplicate.

### 2.5. Cytokine ELISA

BV-2 cells were seeded in 96-well plates (2 × 10^5^/200 *μ*L/well), cultured for 2 days, and incubated with or without LPS (100 ng/mL) in the absence or presence of BFE (25–200 *μ*g/mL) for 24 h. TNF*α* and IL-6 concentrations in supernatants were measured with a commercially available mouse TNF*α* and IL-6 ELISA kits (BioLegend Inc., San Diego), followed by measurements in a plate reader at 540 nm. 

### 2.6. Immunoblotting

Primary microglia were left untreated or treated with LPS (100 ng/mL) in the presence or absence of BFE (25–200 *μ*g/mL) for 24 h. At the end of each experiment, cells were washed with phosphate-buffered saline (PBS) and lysed in 1.3x sodium-dodecyl-sulfate- (SDS-) containing sample buffer without 1, 4-dithio-dl-threitol (DTT) or bromophenol blue containing 100 *μ*M orthovanadate. Protein contents were measured using the bicinchoninic acid (BCA) method (Pierce Protein Biology), according to the manufacturer's instructions. Bovine serum albumin (BSA, Sigma) was used as a standard. Before electrophoresis, a mixture of bromophenol blue and DTT (final concentration, 10 mM) was added to the samples. For western blotting, 40 *μ*g of total protein from each sample was subjected to SDS-PAGE (polyacrylamide gel electrophoresis) under reducing conditions. Proteins were then transferred onto polyvinylidene fluoride (PVDF) membranes (Millipore, Bedford, MA, USA) by semidry blotting. The membranes were blocked for 2 hours at room temperature using Rotiblock (Roth, Karlsruhe, Germany) or nonfat dry milk blotting grade blocker (Biorad, USA) and incubated overnight with primary antibodies. Primary antibodies used were goat anti-COX-2 (Santa Cruz; 1 : 500) and rabbit anti-iNOS (Cell Signalling; 1 : 1000). Primary antibodies were diluted in Tris-buffered saline (TBS) containing 0.1% Tween 20 (TBS-T) and 1% BSA. After extensive washing (three times for 15 min each in TBS-T), COX-2 and iNOS proteins were detected with horseradish peroxidase-coupled rabbit anti-goat IgG (Santa Cruz, 1 : 100,000 dilution) using chemiluminescence (ECL) reagents (Amersham Pharmacia Biotech, Freiburg, Germany). Equal protein loading and transfer were assessed by subjection of each sample to a western blot for actin (rabbit antiactin IgG, diluted 1 : 5000). All western blot experiments were carried out at least three times.

### 2.7. ELISA for NF-*κ*Bp65

SK-N-SH cells were stimulated with IL-1*β* (10 units/mL) in the presence or absence of BFE (25–200 *μ*g/mL) for 1 h. After the stimulation period, nuclear extracts were prepared using Cayman nuclear extraction kit (Cayman Chemical Company, Ann Arbor, USA) according to the manufacturer's instructions. Briefly, cells were collected by scraping and washed twice with cold PBS. Cells were centrifuged for 5 min at 4°C, the supernatant was discarded, and cell pellet was resuspended in 5 mL of ice-cold PBS. The centrifugation procedure was repeated twice and the cell pellets were placed on ice and allowed to swell in 500 *μ*L of 1x hypotonic buffer, followed by addition of 10% NP-40 with gentle mixing. The suspension was centrifuged and the supernatants which contained the cytosolic fractions were stored at −80°C for subsequent analysis of cytoplasmic NF-*κ*B. The pellet was resuspended in 100 *μ*L of ice-cold complete nuclear extraction buffer, vortexed, and rocked gently for 15 min. The samples were then centrifuged and the supernatants (nuclear fractions) were collected. Cytoplasmic and nuclear fractions were measured for levels of NF-*κ*B using human NF-*κ*Bp65 ELISA kit (Invitrogen, California, USA), according to the manufacturer's instructions.

### 2.8. ELISA for I*κ*B Degradation

SK-N-SH cells were left untreated or treated with IL-1*β* (10 units/mL) in the presence or absence of BFE (25–200 *μ*g/mL) for 30 min. At the end of the stimulation period, cells were washed with phosphate-buffered saline (PBS) and lysed with commercially available lysis buffer (New England Biolabs, UK). Cell lysates were subjected to human total I*κ*B ELISA according to the manufacturer's instructions (R&D Systems, Abingdon, UK). Concentrations of total I*κ*B in cell lysates were then measured with a plate reader at 450 nm.

### 2.9. Sandwich ELISA for MAPK Activity

BV-2 microglia were left untreated or treated with LPS (100 ng/mL) in the presence or absence of BFE (25–200 *μ*g/mL) for 30 min. At the end of the stimulation period, cells were washed with phosphate-buffered saline (PBS) and lysed as described for western blot. Cell lysates were subjected to PathScan MAP Kinase Multi-Target Sandwich ELISA for phospho-p38, phospho-42/44, and phospho-JNK according to the manufacturer's instructions (Cell Signalling Technologies). Absorbance values were measured with a plate reader at 450 nm.

### 2.10. MTT Assay for Cell Viability

The viabilities of rat primary microglia and BV-2 microglia after treatment with BFE were determined by the colorimetric 3-(4, 5-dimethylthiazol-2-yl)-2, 5-diphenyl tetrazolium bromide (MTT) assay. The yellow compound MTT is reduced by mitochondrial dehydrogenases to the water-insoluble blue compound formazan, depending on the viability of cells. Primary microglia and BV2 microglia were incubated with or without LPS (100 ng/mL) in the absence or presence of BFE (25–200 *μ*g/mL) for 24 h. Twenty microlitres of MTT solution (Sigma) (5 mg/mL) was added to each well. The plate was incubated for 4 h at 37°C in a CO_2_-incubator. One hundred and eighty microlitres of medium was removed from every well without disturbing the cell clusters. A 180 *μ*L methanol/DMSO solution (50 : 50) was added to each well, and the preparations were mixed thoroughly on a plate shaker with the cell containing formazan crystals. After all of the crystals were dissolved, the absorbance was read at 540 nm with a microplate reader.

### 2.11. Statistical Analysis

Values of all experiments were represented as mean ± SEM of at least 3 experiments. Values were compared using *t*-test (two groups) or one way ANOVA with post-hoc Student Newman-Keuls test (multiple comparisons). The level of significance was set at *P* < 0.05.

## 3. Results and Discussion 

### 3.1. BFE Reduced PGE_**2**_, Nitrite, and Proinflammatory Cytokine Production from LPS-Stimulated Microglia

**Figure 1:**
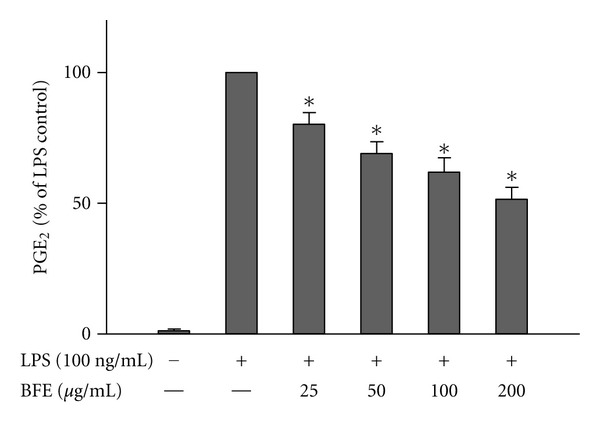
Reduction of PGE_2_ production in LPS-stimulated microglia. Cells were stimulated with LPS (100 ng/mL) in the presence or absence of BFE (25–200 *μ*g/mL) for 24 h. At the end of the incubation period, supernatants were collected for PGE_2_ measurement. All values are expressed as mean ± SEM for 3 independent experiments. Data were analysed using one-way ANOVA for multiple comparison with post-hoc Student Newman-Keuls test. *P < 0.05 in comparison with LPS control.

**Figure 2:**
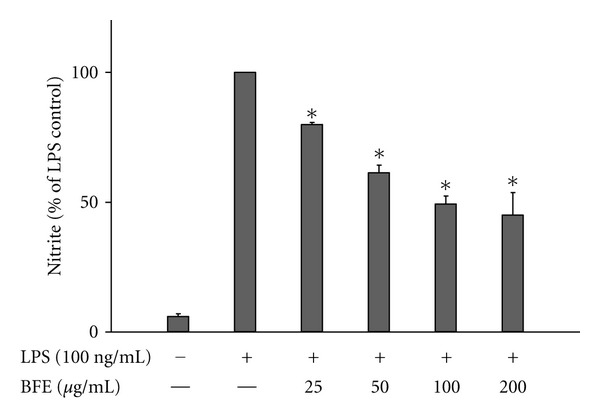
Reduction of nitrite production in LPS-stimulated microglia. Cells were stimulated with LPS (100 ng/mL) in the presence or absence of BFE (25–200 *μ*g/mL) for 24 h. At the end of the incubation period, supernatants were collected for nitrite measurement with the Griess assay. All values are expressed as mean ± SEM for 3 independent experiments. Data were analysed using one-way ANOVA for multiple comparison with post-hoc Student Newman-Keuls test. *P < 0.05 in comparison with LPS control.

**Figure 3:**
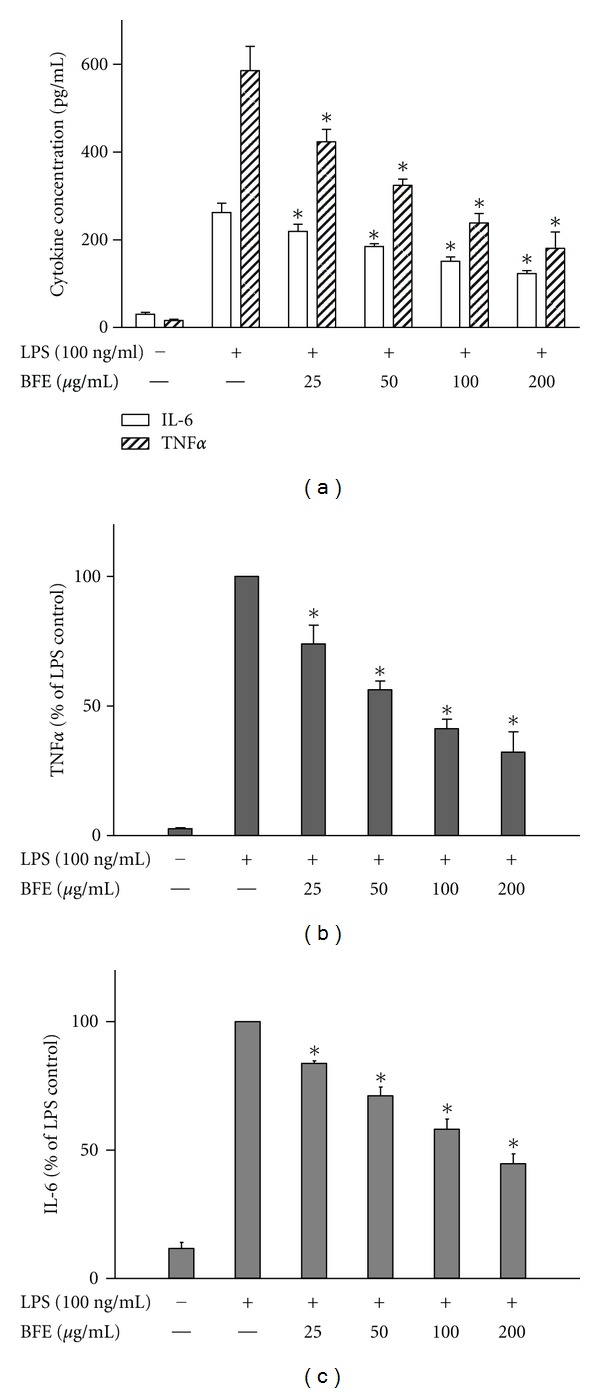
Reduction of TNF*α* ((a) and (b)) and IL-6 ((a) and (c)) production in LPS-stimulated BV-2 microglia. Cells were stimulated with LPS (100 ng/mL) in the presence or absence of BFE (25–200 *μ*g/mL) for 24 h. At the end of the incubation period, supernatants were collected for TNF*α* and IL-6 measurement according to the manufacturer's instructions. All values are expressed as mean ± SEM for 3 independent experiments. Data were analysed using one-way ANOVA for multiple comparison with post-hoc Student Newman-Keuls test. *P < 0.05 in comparison with LPS control.

Following release from activated microglia, NO, PGE_2_, and proinflammatory cytokines such as TNF*α* have been implicated as important mediators in the processes of central nervous system (CNS) inflammation, which is associated with neurodegenerative diseases [17]. To investigate whether BFE interferes with these mediators, experiments were carried out to determine its effects on their production in LPS-stimulated BV-2 microglia. Cells were pre-treated with BFE, followed by stimulation with LPS (100 ng/mL) for 24 h. Results show that incubation of cells with LPS alone for 24 h resulted in an increase in PGE_2_ level in supernatants (Figure 1). However, pretreatment with BFE (25–200 *μ*g/mL) prior to LPS stimulation resulted in significant (*P* < 0.05) reduction in PGE_2_. A similar trend was observed with supernatants analysed for nitrite production using the Griess assay (Figure 2) and ELISA measurements for TNF*α* and IL-6 (Figures 3(a) and 3(b)). The highest concentration of BFE used in this experiments (200 *μ*g/mL) reduced PGE_2_, nitrite, TNF*α*, and IL-6 production in LPS-stimulated BV-2 microglia by 51.6, 45, 32.2, and 44.7%, respectively, demonstrating that BFE strongly inhibits these factors in LPS-induced neuroinflammation. Although *B. ferruginea* has been widely reported to exhibit anti-inflammatory effects *in vivo* [5–7], the exact mechanism(s) involved were unknown. Our current results show that inhibition of PGE_2_, nitric oxide, and the proinflammatory cytokines plays significant roles in the earlier observed *in vivo* actions of *B. ferruginea*. A cell viability assay carried out at the same time revealed that BFE did not affect viability of BV-2 microglia at concentrations which produced reductions in PGE_2_, nitrite and cytokines produced from the cells (data not shown).

### 3.2. BFE Effects Are Mediated through Inhibition of COX-2 and iNOS Protein Expression

**Figure 4:**
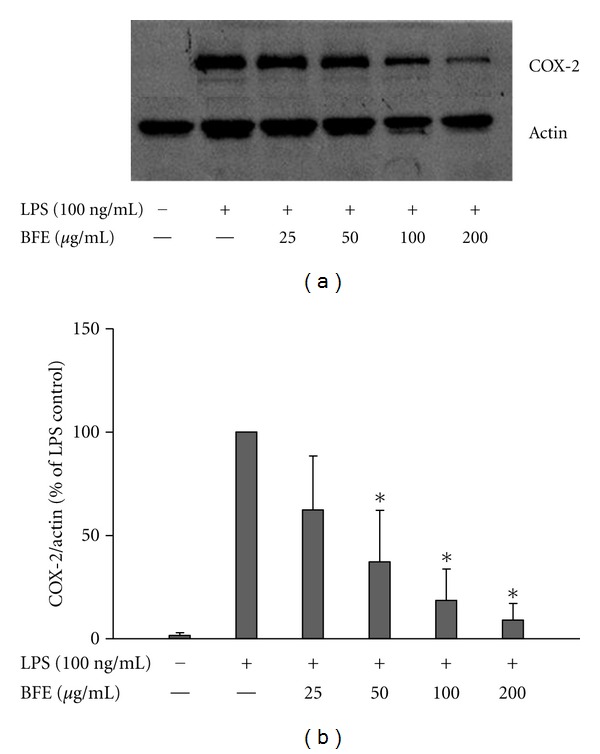
(a) BFE inhibited COX-2 protein expression in LPS-stimulated primary microglia. Cells were stimulated with LPS (100 ng/mL) in the presence or absence of BFE (25–200 *μ*g/mL) for 24 h. At the end of incubation period, COX-2 protein levels were measured using western blot using specific antibodies for each protein. (b) Quantitative densitometric analysis of COX-2 protein expression normalized to actin loading control. All values are expressed as mean ± SEM for 3 independent experiments. Data were analysed using one-way ANOVA for multiple comparison with post-hoc Student Newman-Keuls test. *P < 0.05 in comparison with LPS control.

Following our observation that BFE produced marked effects on PGE_2_ and NO, we hypothesised that it might be targeting NF-*κ*B, and thus interfere with the transcription of NF-*κ*B-regulated genes like COX-2 and iNOS [18]. Western blot experiments were carried out to investigate the effects of BFE on COX-2 and iNOS proteins. Rat primary microglia cells were stimulated with LPS (100 ng/mL) for 24 h; this was followed by a marked increase in COX-2 protein. Results showed that BFE produced a dose-dependent and significant (*P* < 0.05) reduction in COX-2 protein immunoreactivity induced by LPS (Figure 4).

**Figure 5:**
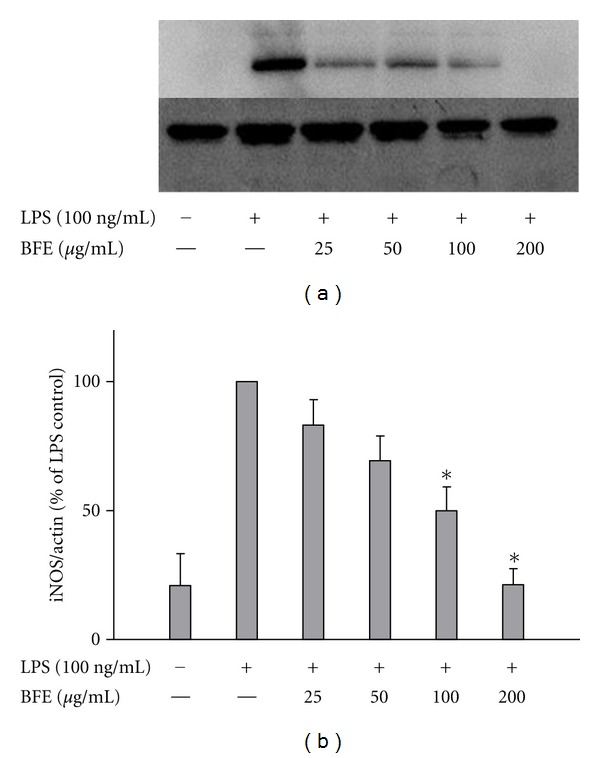
(a) BFE inhibited iNOS protein expression in LPS-stimulated primary microglia. Cells were stimulated with LPS (100 ng/mL) in the presence or absence of BFE (25–200 *μ*g/mL) for 24 h. At the end of incubation period, iNOS protein levels were measured using western blot using specific antibodies for each protein. (b) Quantitative densitometric analysis of iNOS protein expression normalized to actin loading control. All values are expressed as mean ± SEM for 3 independent experiments. Data were analysed using one-way ANOVA for multiple comparison with post-hoc Student Newman-Keuls test. *P < 0.05 in comparison with LPS control.

Experiments also showed that BFE produced a dose-dependent and significant inhibition of iNOS protein expression in LPS-stimulated primary microglia (Figure 5). These results are consistent with and confirm that the reduction in PGE_2_ and NO production by BFE are mediated through the inhibition of COX-2 and iNOS protein expressions, respectively.

### 3.3. BFE Interferes with Nuclear Translocation of NF-*κ*Bp65

**Figure 6:**
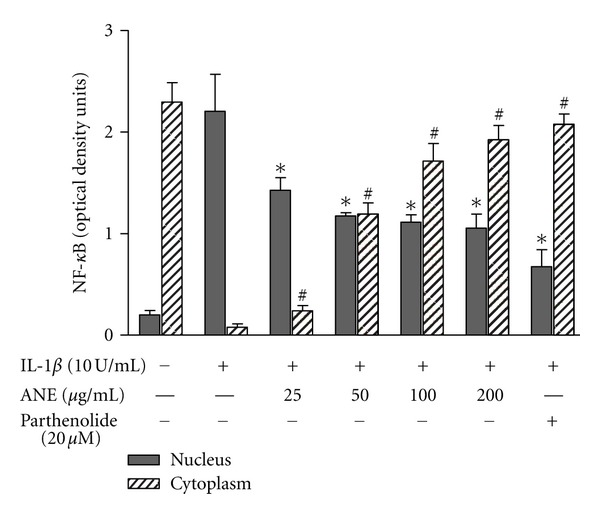
BFE inhibited IL-1*β*-induced nuclear translocation of NF-*κ*B. SK-N-SH cells were stimulated with IL-1*β* in the presence or absence of BFE (25–200 *μ*g/mL) for 1 hour. Cell lysates (nuclear and cytoplasmic extracts) were then analysed for total NF-*κ*B using human NF-*κ*Bp65 ELISA kit. All values are expressed as mean ± SEM for 3 independent experiments. Optical densities were measured at 450 nm with a microplate reader. Data were analysed using one-way ANOVA for multiple comparison with post-hoc Student Newman-Keuls test. ^∗, #^ *P* < 0.05 in comparison with IL-1*β* control.

On dissociating from I*κ*B, NF-*κ*B moves to the nucleus to trigger the expression of multiple proinflammatory genes, including COX-2 and iNOS. To further investigate whether BFE interfered with nuclear translocation of NF-*κ*B, we employed an ELISA to measure cytoplasmic and nuclear levels of the p65 subunit of NF-*κ*B in SK-N-SH cells, in the presence and absence of the extract. IL-1*β* treatment caused a marked reduction in cytosolic, and an increase in nuclear p65 levels when compared with unstimulated cells (Figure 6). However, pretreatment with BFE (25–200 *μ*g/mL) resulted in a statistically significant (*P* < 0.05) increase in cytosolic and decrease in nuclear NF-*κ*Bp65, thereby effectively inhibiting nuclear translocation of the subunit from the cytoplasm to the nucleus. In this experiment, parthenolide (20 *μ*M) was used as reference compound. Parthenolide is a known NF-*κ*B inhibitor which acts by targeting I*κ*B kinase complex [19, 20] and has been shown to inhibit inflammatory mediators such as IL-6, TNF*α*, IL-1*β*, IL-12 [21], PGE_2_ [22, 23], and NO/iNOS [24] in various cells. Parthenolide exhibited the strongest inhibition of NF-*κ*B activation against IL-1*β*-induced NF-*κ*B activation and nuclear translocation in this experiment.

### 3.4. BFE Interferes with NF*κ*B by Inhibiting I*κ*B Degradation

**Figure 7:**
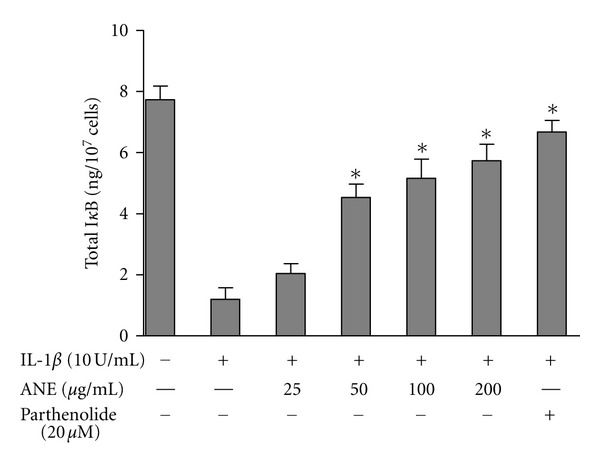
Effects of BFE on I*κ*B degradation in IL-1*β*-stimulated SK-N-SH cells. Cells were stimulated with IL-1*β* in the presence or absence of BFE (25–200 *μ*g/mL) for 30 minutes. Cell lysates were then evaluated for I*κ*B degradation by measuring the amount of total I*κ*B in cell lysates. Data were analysed using one-way ANOVA for multiple comparison with post-hoc Student Newman-Keuls test. *P < 0.05 in comparison with IL-1*β* control.

To further understand the molecular mechanism of action of BFE, and to investigate the role of I*κ*B in its actions, we evaluated its action on I*κ*B degradation in SK-N-SH neuroblastoma cells. IL-1*β* resulted in complete degradation of I*κ*B*α* in these cells. BFE (50–200 *μ*g/mL) significantly (*P* < 0.05) blocked IL-1*β*-induced degradation of I*κ*B*α* (Figure 7), indicating that the subsequent inhibition of NF-*κ*B activation was due to the influence of BFE on I*κ*B*α* by BFE. Parthenolide (20 *μ*M) almost completely reversed LPS-induced degradation of I*κ*B in SK-N-SH cells.

### 3.5. BFE Inhibited LPS-Induced Phosphorylation of p38, but Not p42/44 and JNK

**Figure 8:**
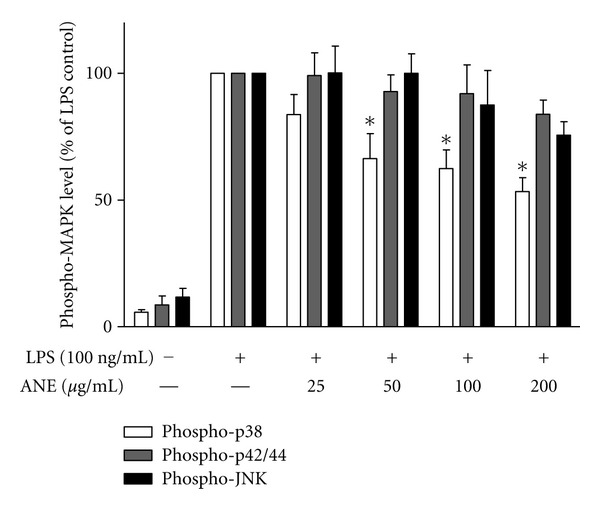
BFE inhibited p38, but not p42/44 or JNK MAPK in LPS-stimulated BV-2 microglia. Lysates from LPS-stimulated microglia were analysed using ELISA for phospho-p38, phospho-42/44, and phospho-JNK. Data were analysed using one-way ANOVA for multiple comparison with post-hoc Student Newman-Keuls test. *P < 0.05 in comparison with LPS control.

p38 MAP kinase (MAPK) participates in a signalling cascade controlling the cellular response to proinflammatory cytokines and a variety of cellular stresses. The p38 MAPK signalling has also been widely accepted as a cascade contributing to neuroinflammation [12]. ERK and p38 MAPK cascades have been reported to contribute to regulation of iNOS and TNF*α* gene expression in LPS-activated glial cells [25]. We therefore investigated whether anti-inflammatory effects of BFE might be related to the inhibition of MAPK phosphorylation in BV-2 cells activated with LPS. Results show that LPS treatment caused marked phosphorylation of p38, p42/44, and JNK MAP kinases (Figure 8) in BV-2 cells. Pretreatment with BFE did not produce significant effects on the phosphorylation of p42/44 or JNK due to LPS stimulation. However, at 50–200 *μ*g/mL, BFE produced statistically significant (*P* < 0.05) and dose-related inhibition of p38 phosphorylation by LPS. The p38 MAPK signalling pathway has been widely reported to activate NF-*κ*B and the subsequent induction of proinflammatory genes [26–28]. We postulate that BFE might be exerting an inhibitory action on NF-*κ*B through p38-dependent action.

## 4. Conclusions

In this first attempt at elucidating the molecular targets of anti-inflammatory actions of *B. ferruginea*, we have established that an extract of this plant inhibited the production of inflammatory cytokines, PGE_2_ and NO following inflammatory stimulation. These actions are probably related to the action of the extract on p38-mediated NF-*κ*B proinflammatory gene transcription or I*κ*B-mediated activation and nuclear translocation NF-*κ*B. These results further validate the use of B. ferruginea in TAM for treating inflammatory conditions. The observed anti-neuroinflammatory actions of BFE suggest that this plant might be a potential source of new therapeutic substances for neuroinflammatory/neurodegenerative conditions, such as Alzheimer's disease. Experiments are in progress to isolate the active components in this plant.
